# Perceived Neighborhood Characteristics and Cognitive Functioning among Diverse Older Adults: An Intersectional Approach

**DOI:** 10.3390/ijerph18052661

**Published:** 2021-03-06

**Authors:** Amy D. Thierry, Kyler Sherman-Wilkins, Marina Armendariz, Allison Sullivan, Heather R. Farmer

**Affiliations:** 1 Department of Public Health Sciences, Xavier University of Louisiana, New Orleans, LA 70125, USA; asulliva@xula.edu; 2 Department of Sociology and Anthropology, Missouri State University, Springfield, MO 65897, USA; kshermanwilkins@MissouriState.edu; 3 Department of Biobehavioral Health, The Pennsylvania State University, University Park, PA 16802, USA; mpa5220@psu.edu; 4 Department of Human Development and Family Sciences, University of Delaware, Newark, DE 19716, USA; hfarmer@udel.edu

**Keywords:** cognitive functioning, neighborhoods, intersectionality, older adults, health disparities

## Abstract

Unfavorable neighborhood conditions are linked to health disparities. Yet, a dearth of literature examines how neighborhood characteristics contribute to cognitive health in diverse samples of older adults. The present study uses an intersectional approach to examine how race/ethnicity, gender, and education moderate the association between neighborhood perceptions and cognitive functioning in later life. We used data from adults ≥65 years old (*n* = 8023) in the 2010–2016 waves of the nationally representative Health and Retirement Study (HRS). We conducted race/ethnicity-stratified linear regression models where cognitive functioning, measured using the 35-point Telephone Interview Cognitive Screen (TICS), was regressed on three neighborhood characteristics—cleanliness, safety, and social cohesion. We examine whether there is heterogeneity within race/ethnicity by testing if and how the relationship between neighborhood characteristics and cognitive functioning differs by gender and education. Among White adults, worse neighborhood characteristics were associated with lower cognitive functioning among those with less education. However, for Black adults, poor perceived quality of one’s neighborhood was associated with worse cognitive functioning among those with more years of education compared to those with fewer years of education. Among Mexicans, perceived neighborhood uncleanliness was associated with lower cognitive functioning among those with less education, but higher cognitive functioning for those with higher levels of education. Thus, this study contributes to the literature on racial/ethnic disparities in cognitive aging disparities by examining neighborhood contextual factors as determinants of cognitive functioning. In particular, we find that higher education in the context of less favorable neighborhood environments does not confer the same benefits to cognitive functioning among all older adults.

## 1. Introduction

Extensive research demonstrates that unfavorable and disadvantaged neighborhood conditions, such as those characterized by low socioeconomic status (SES), limited health care access, and other poor social and economic factors, contribute to health disparities [1,2,3,4]. In particular, racial/ethnic minorities disproportionately reside in disadvantaged neighborhoods and face unequal rates of chronic conditions, such as obesity, diabetes, and hypertension [5]. Given that these conditions have also been linked to cognitive declines in later life, research is needed to explore how neighborhood conditions may be associated with disparities in cognitive functioning [6,7]. While studies have examined the relationship between objective socioeconomic and physical environment factors at the neighborhood level and various measures of cognition, findings are mixed [8]. Literature suggests that neighborhood perceptions, including perceived disorder and social cohesion, may have consequences on health through both stress and behavioral pathways [9]. Moreover, previous research shows that Black and Hispanic adults endorse worse neighborhood quality related to safety, cleanliness, and cohesion compared to Whites [9,10,11]. Yet, a dearth of literature examines how perceived neighborhood characteristics—that is, how residents rate their neighborhoods—contribute to cognitive functioning among diverse samples of US older adults. Thus, the purpose of the present study is to examine how the association between neighborhood perceptions and cognitive functioning among older adults varies by race/ethnicity, gender, and SES.

### 1.1. Disparities in Cognitive Function

Cognitive functioning in older adults can change with normal aging; however, cognitive declines, such as dementia and cognitive impairment, are not considered normative parts of the aging process. Evidence suggests disparities in poor cognitive functioning are patterned by race/ethnicity and social factors, including SES and gender [7]. For example, disparities in Alzheimer’s disease and related dementias (ADRD) in the US have been documented, where, compared to Whites, the prevalence of ADRD is twice as high in African Americans and 1.5 times greater among Hispanics [12,13]. Women compared to men as well as adults with lower educational attainment are disproportionately burdened by ADRD, though less is known about how race/ethnicity may structure these disparities [13]. Given the consequential implications of ADRD diagnoses for older adults, research has aimed to understand patterns in cognitive functioning indicators that may increase risk for ADRD. Still, disparities in cognitive function outcomes, including memory, attention, and reasoning, are apparent [14,15].

From both a life course perspective and minority stress framework, older racial and ethnic minorities may face lower SES and worse social conditions (e.g., segregated, disordered neighborhoods lacking access to valuable social resources) which can increase stress, thereby contributing to cognitive decline [16]. An established literature supports that neighborhoods are important for the health of older adults, with neighborhood-level SES being the strongest predictor of physical health [17]. Yet, there is a gap in understanding how social determinants, such as neighborhood environment, may be implicated in cognitive outcomes.

### 1.2. Neighborhoods and Cognition

Evidence suggests that neighborhood characteristics are associated with cognition in older adults [18]. For example, previous studies show that neighborhood socioeconomic disadvantage is a risk factor for lower cognitive functioning among older adults, especially among individuals with low SES [19,20,21]. That is, residing in a neighborhood with limited health-enabling built resources (i.e., poor walkability) have been linked to accelerated cognitive decline among older adults [18]. However, studies that have examined how physical and social aspects of the neighborhood environment are linked to cognitive functioning yield mixed findings. Lee and colleagues (2011) investigated whether increased exposure to psychosocial risks, such as social disorganization, physical disorder, public safety, and economic deprivation, were associated with worse cognitive functioning for urban residents aged 50 to 70. They found that worse neighborhood-level factors were associated with poor cognitive performance only among residents with the APOE e4 genotype, a strong predictor of risk for Alzheimer’s disease [22].

Additionally, recent knowledge posits that perceived neighborhood characteristics may influence cognition by causing heightened psychological distress [23]. For instance, racial/ethnic minorities living in segregated neighborhoods experience higher rates of poverty and its associated negative conditions such as crime and deteriorating infrastructure [7]. These characteristics are likely linked to poor cognitive functioning in part because of the chronic stress these exposures produce [7,24]. Nevertheless, few studies to date have examined how perceptions of neighborhood characteristics, including negative perceptions of safety, cleanliness, and social cohesion, differentially are associated with cognition at the intersection of race/ethnicity, gender, and SES.

### 1.3. Intersectionality

As the aging population is expected to become more racially and ethnically diverse and female [25], it is imperative to understand how social inequalities contribute to population health disparities in later life. Social factors, such as race/ethnicity, SES, and gender, often intersect, creating differential access to resources and exposure to risks, thereby resulting in health inequalities for disenfranchised groups. Indeed, some research shows that Black and Mexican mid-life and older adults experience steeper health declines with age [26], including worse cognitive outcomes for Black men, as well as foreign-born Hispanic men and women, relative to non-Hispanic Whites [27]. Access to socioeconomic resources, such as higher educational attainment, is linked to better cognitive health [28]. Research suggests that education may be particularly protective for cognitive outcomes: older adults residing in low SES neighborhoods had lower cognitive functioning but there was no association between living in a low SES neighborhood and cognitive functioning among those with high educational attainment [29]. Examinations of the interactive relationships of race/ethnicity, gender, and education in the context of neighborhood environments and cognition are limited.

Although disparities by race/ethnicity, gender, or SES are well-documented, there is growing interest in examining the intersection of these factors in order to better understand and address disparities [30]. Scholars highlight the utility of intersectionality in population health studies to improve our understanding of social and structural/systemic processes contributing to persistent health disparities for racial/ethnic minorities, women, and individuals with low SES [30,31,32,33]. For example, prior research points to a link between low individual SES and neighborhood-level exposures, including concentrated disadvantage and social disorder, which were subsequently linked to poor health outcomes [34]. Intersectionality, grounded in Black feminist theory [35], offers a framework for contextualizing population health disparities that examines the combined stratification of multiple marginalized identities, social positions, and/or unfair structural determinants, such as neighborhood environments. However, the application of intersectionality theory to health has focused more on physical health conditions rather than cognition. The existing body of research does not consider the possible ways in which the relationship between perceived neighborhood characteristics and cognition in later life may differ across race/ethnicity, SES, and gender.

The purpose of this study is to address previous gaps in the literature by assessing whether the relationship between neighborhood perceptions and cognitive functioning is patterned by race/ethnicity, gender, and education in a nationally representative sample of US older adults. Our research questions are as follows:

(1) Are perceived neighborhood characteristics associated with cognitive functioning among older US adults?
(2) Is the association between perceived neighborhood characteristics and cognitive functioning moderated by race/ethnicity, gender, and education?

We hypothesize that neighborhood perceptions (uncleanliness, unsafety, and low social cohesion) will be associated with worse cognitive functioning. We further hypothesize that the relationship between perceived neighborhood characteristics and cognitive functioning will vary across the intersections of race/ethnicity, education, and gender.

## 2. Materials and Methods

### 2.1. Sample

We used data from the 2010–2016 waves of the nationally representative Health and Retirement Study (HRS). Data are derived from the HRS Core Survey, Psychosocial Leave-Behind questionnaire, and RAND imputed data file. Our analytic sample includes 8023 unique non-Hispanic White, non-Hispanic Black, and Mexican American respondents ages 65 and older. We limit our analyses to adults who are 65 years of age and older because the cognitive functioning measure of interest is acquired from HRS participants ages 65+. Given extensive heterogeneity in the Hispanic population, we limit the sample to only Mexican-descent older adults. Other Hispanic groups were unable to be examined in stratified analyses due to small sample sizes. It is important to note that a focus on Mexican Americans is appropriate given that this population represents the largest segment of the Hispanic population in the US, as well as the fastest growing ethnic group.

### 2.2. Measures

#### 2.2.1. Cognitive Functioning

To measure cognitive functioning, we used the 35-point Telephone Interview Cognitive Screen (TICS). The TICS is a well-established composite cognition score which has previously been used to assess cognitive functioning of older adults [36,37]. The score is based on HRS participants’ performance on several tasks including naming, serial subtraction, backward count, and both immediate and delayed word recall. All cognitive tasks were conducted as part of the HRS data collection. For the naming task, respondents were tasked with identifying the object used to cut paper (scissors), the name of the prickly plant that grows in the desert (cactus), the name of the current President and Vice President, and the date (day, month, year, and day of the week). The scores on this task ranged from 0–8, with higher scores indicating more correct responses. Serial subtraction called for respondents to subtract seven from 100 and to continue subtracting seven from the previous difference over a total of five trials. Scores ranged from 0–5 and reflect the number of correct subtractions. For the backwards count, the interviewers tasked the participants with counting backwards from twenty as quickly as possible. Scores range from 0–2, with a score of ‘0’ denoting failure in completing the task over two attempts, ‘1’ if the respondent was successful in the second attempt, and ‘2’ if the respondent was successful in the first attempt. Lastly, the immediate and delayed recall required participants to recall ten words given to them during the interview. For the immediate recall task, respondents were asked to repeat words right after they were given, while the delayed recall task required respondents to recall the words after a five-minute delay. Scores for each task ranged from 0–10, with higher scores denoting more words recalled. To calculate the composite TICS score, all scores for the aforementioned cognitive tasks were summed (range: 0–35), with higher scores indicating better cognitive functioning. The composite TICS score was provided within the HRS RAND data file.

#### 2.2.2. Neighborhood Perceptions

HRS participants responded to questions in the Psychosocial Leave-Behind questionnaire about their perceptions of neighborhood disorder and social cohesion. Neighborhood disorder, categorized as safety and cleanliness, was determined by participants’ rating of the following survey items on a scale from 1 = highly agree to 7 = highly disagree. Perceived safety was assessed through two items that measured whether respondents felt safe to walk alone after dark and whether their neighborhood had vandalism and graffiti. Perception of cleanliness was measured using two items: (1) this area is kept very clean and (2) there are no vacant/deserted houses. Social cohesion was assessed by participants’ responses to the following survey questions on a scale from 1 = highly agree to 7 = highly disagree: (1) feel a part of this area, (2) most people can be trusted, (3) most people are friendly, and (4) people help you if in trouble. To create variables for perceived safety, cleanliness, and social cohesion, scores for each question were summed and averaged. Thus, higher scores for each variable represent worse perceptions of the corresponding neighborhood characteristic.

#### 2.2.3. Demographic Characteristics

Information on demographic characteristics were obtained from the HRS Core survey data files. Race/ethnicity was categorized as non-Hispanic White (hereafter referred to as White), non-Hispanic Black (hereafter referred to as Black), and Mexican. Gender (1 = woman), nativity (1 = foreign-born), and marital status (1 = married or partnered) were coded as dichotomous variables. Educational attainment was measured continuously as number of years of schooling completed (range: 0–17). Age, household income, and wealth were measured continuously. Household income and wealth were both natural log transformed due to non-normal distributions.

#### 2.2.4. Health Characteristics

Physical health status was assessed by self-reported count of chronic conditions, including high blood pressure, diabetes, cancer, lung disease, heart disease, stroke, and arthritis (range: 0–7). Individuals with a BMI ≥30 were classified as obese. As depressive symptoms may modify cognitive functioning, a dichotomous variable indicating depression was created based on the Center for Epidemiologic Studies Depression (CES-D) scale (range: 0–8) using an established cutoff for depression (≥3) [38]. Behavioral risk factors included smoking status (coded as 0 = never smoker, 1 = former smoker, and 2 = current smoker), alcohol use (coded as 0 = never drinks alcohol, 1 = drinks 1–2 drinks on days when drinking, and 2 = 3 or more drinks per day when drinking) and respondents’ engagement in moderate to vigorous physical activity (coded as 0 = hardly ever or never, 1 = sometimes [once a week or one to three times a month], and 2 = frequently [at least once per week]).

### 2.3. Analytic Strategy

We calculated weighted descriptive statistics across all variables for the entire sample and stratified by race/ethnicity. To test whether Black and Mexican older adults differed from Whites across our variables of interest we estimated a series of unadjusted linear and logistic regression models (as appropriate) with Whites serving as the reference category.

To examine the relationship between each perceived neighborhood characteristic and cognitive functioning across race/ethnicity, we estimated a series of weighted race/ethnicity-stratified linear multiple regression models. These models regressed cognitive functioning on each perceived neighborhood characteristic (safety, cleanliness, and social discohesion) separately. All models controlled for age, gender, education, nativity, income, wealth, marital status, CES-D, smoking status, alcohol consumption, physical activity, obesity, and a count of chronic conditions. Statistical significance was determined at *p* < 0.05. As we were interested in understanding whether gender and education moderated the relationship between each perceived neighborhood characteristic and cognitive functioning across race/ethnicity, we re-estimated the aforementioned regression models for each neighborhood characteristic with neighborhood characteristic x (1) gender and (2) education interaction terms. Lastly, we plotted statistically significant (*p* < 0.1) interaction terms to aid in our interpretation of the moderating role of both gender and education by race/ethnicity. Kernel density functions for each regression model demonstrated normal distribution of the residuals. All analyses were conducted using Stata 16/SE (StataCorp, College Station, TX, USA).

## 3. Results

### 3.1. Descriptive Statistics

Means and proportions for all variables both for the entire sample and stratified by race/ethnicity can be found in Table 1. Relative to Whites, both Black and Mexican American adults had lower levels of cognitive functioning. Moreover, Blacks and Mexican Americans had worse perceptions of their neighborhoods in terms of safety, cleanliness, and social cohesion compared to their White peers. Black and Mexican American older adults on average had lower SES (education, income, and wealth) than Whites. For example, there were stark differences in education, where White older adults had 13.28 years of education (SE = 0.06), compared to 11.92 years (SE = 0.12) in Black adults and 8.66 years (SE = 0.37) in Mexican American adults. Racial/ethnic differences were also apparent in behavioral risk factors and chronic disease burden, with Blacks more likely than Whites to be current smokers, obese, and have a higher number of chronic conditions. Both Black and Mexican American adults were more likely to report depressive symptoms than Whites. It is important to note that fewer Black and Mexican American adults consumed alcohol relative to Whites. Finally, while Whites were more likely than Blacks and Mexican Americans to frequently engage in moderate/vigorous physical activity, Black and Mexican adults were more likely to report sometimes participating in moderate/vigorous physical activity.

### 3.2. Regression Analyses

**Figure 1:**
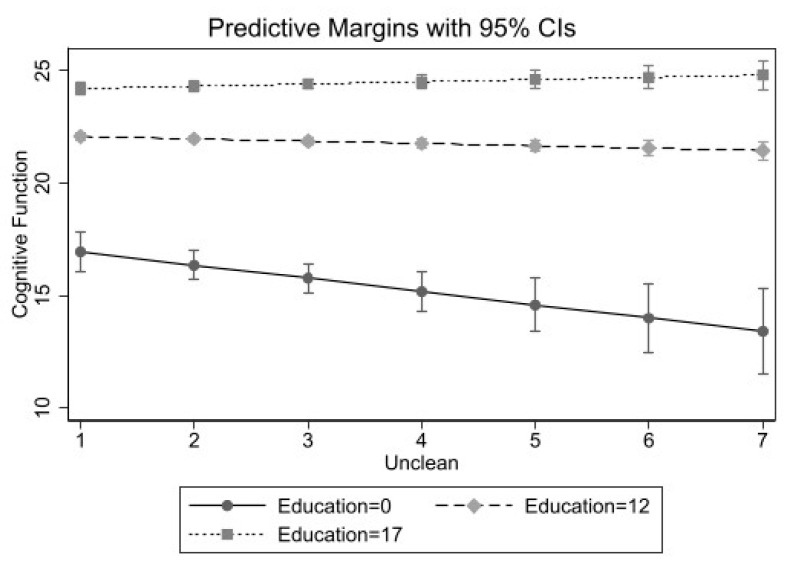
Perceived Unclean Neighborhood by Years of Education Predicting Cognitive Functioning among Non-Hispanic White Older Adults (*n* = 6616).

Table 2, Table 3 and Table 4 present the fully adjusted regression models stratified by race/ethnicity with interaction terms included. Across all racial/ethnic groups, gender did not moderate the relationship between any of the neighborhood characteristics and cognitive functioning. Results for Whites (Table 2) indicate significant interactions between years of education and each perceived neighborhood characteristic. Figure 1 demonstrates the interaction between education and unclean neighborhood on cognitive functioning for Whites. Results indicate that the relationship between perceived neighborhood uncleanliness and lower cognitive functioning was stronger for those with lower levels of education. This is shown by the steeper negative slope for the solid line which corresponds to those with lower education levels. Plots of the remaining interactions are comparable (see Supplementary Materials).

**Figure 2:**
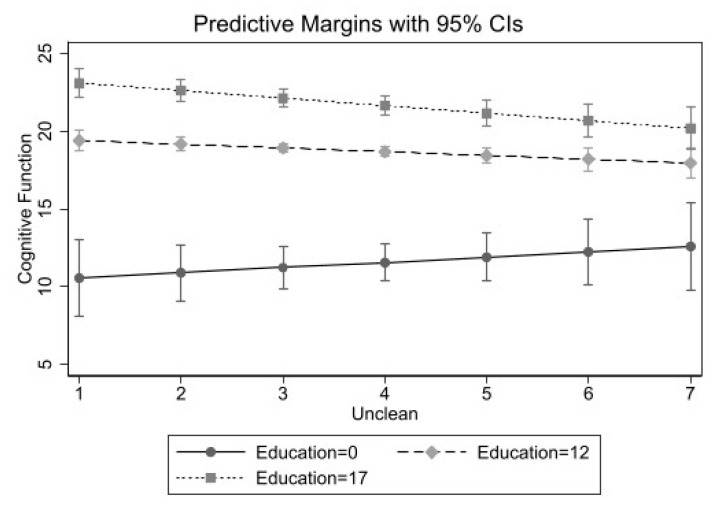
Perceived Unclean Neighborhood by Years of Education Predicting Cognitive Functioning among Non-Hispanic Black Older Adults (*n* = 1044).

Table 3 presents estimates from fully adjusted models including interaction terms for neighborhood characteristic x gender and neighborhood characteristic x education among Black older adults. Similar to Whites, we only found statistically significant interactions between each neighborhood characteristic and years of education. As shown in Figure 2, there was a different pattern in how education shaped the relationship between a perceived unclean neighborhood and cognitive functioning among Black older adults. Among those with lower education, perceiving one’s neighborhood as unclean was associated with better cognitive function. As shown, the solid line corresponding to lower education level indicates a positive slope. Moreover, Black older adults with higher levels of education who reported unclean neighborhood conditions had lower levels of cognitive functioning (shown in the dashed lines). Similar patterns for perceived lack of safety and social cohesion were found as those presented for uncleanliness. These figures are available in the Supplementary Materials.

**Figure 3:**
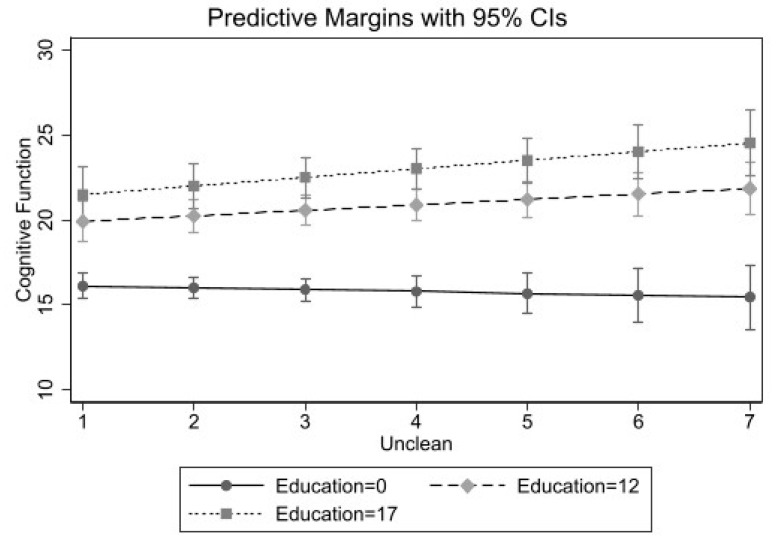
Perceived Unclean Neighborhood by Years of Education Predicting Cognitive Functioning among Mexican Older Adults (*n* = 363).

Lastly, Table 4 shows estimates from linear regression models for Mexican Americans. There was only a statistically significant interaction between one’s perception of the neighborhood as unclean and years of education on cognitive functioning. Figure 3 shows that Mexican Americans with lower education had slightly lower cognitive functioning when they perceived their neighborhoods as unclean. However, Mexican American adults with higher levels of education who perceived their neighborhood as unclean had higher cognitive functioning scores as indicated by the dashed lines.

## 4. Discussion

In this study, we aimed to examine the relationship between perceived neighborhood characteristics (safety, cleanliness, and social cohesion) and cognitive functioning in a nationally representative study of older US adults. We tested two hypotheses in order to expand extant knowledge on social determinants of cognitive inequities. First, our hypothesis that older adults who reported worse neighborhood perceptions would have worse cognitive functioning was supported. We found that worse ratings of safety, cleanliness, and social cohesion were associated with lower cognitive functioning. Next, using intersectionality as a guiding framework [35], we hypothesized that the relationship between neighborhood perceptions and cognitive functioning would differ by race/ethnicity, gender, and education. Consistent with intersectionality theory, the data are indicative of heterogeneity in how social statuses combine to modify the association between neighborhood characteristics and cognitive functioning. We found that neighborhood perceptions interacted with education but not gender; however, this association differs across race/ethnicity. Among White older adults with lower levels of education, there was a negative association between worse neighborhood perceptions and cognitive functioning. Thus, cognitive functioning of more highly educated White older adults was less likely to be affected by living in a less favorable neighborhood environment. Results are similar for Mexican American adults, except that the interaction between neighborhood characteristics and education was only significant for uncleanliness. There was a less pronounced negative association with cognitive functioning among Mexican adults with lower education who perceived their neighborhoods negatively compared to Whites. Among Black older adults, we find the opposite relationship between neighborhood perceptions and cognitive functioning by years of education. Older Black adults with fewer years of education had better cognitive functioning even when they reported residing in less favorable neighborhoods. Conversely, among older Black adults with more education, negative perceptions of neighborhood characteristics were associated with lower cognitive functioning. We did not find statistically significant interactions between neighborhood characteristics and gender within any racial/ethnic group.

The results for Whites are in line with previous theoretical and empirical work showing that higher SES is protective for health [39,40,41,42,43]. Similarly, recent research has shown that upward socioeconomic mobility is associated with better cognitive functioning among Mexican Americans [28]. However, higher educational attainment was not protective against negative neighborhood perceptions among Black adults. Such differential outcomes demonstrate the importance of taking an intersectional approach to understand unique contexts and consequences, particularly for older adults [26,42]. While previous research has shown that educational attainment partially explains racial disparities for Black adults in episodic memory and executive functioning [44], our findings for Black adults warrant alternative explanations. For example, the differential impact of education for Blacks compared to other racial/ethnic groups may be the result of racial residential segregation, even among higher SES Blacks [45,46]. Some cognitive research suggests that social vigilance, or the frequent scanning of one’s environment for threats, in high stress environments may be a source of resiliency against negative cognitive outcomes [47]. As such, adverse neighborhood characteristics which are disproportionately experienced by low SES Blacks may not always lead to worse cognitive functioning in older age. Additionally, cognitive reserve—the capacity to draw on protective cognitive resources—has been proposed as a mechanism to explain why some older adults maintain healthy cognitive functioning even if underlying pathologies are present [48,49]. Thus, cognitive reserve may play a role in preserving cognitive functioning among low SES Black older adults who live in neighborhoods with high disorder or low social cohesion. For example, heterogeneity in the educational experiences among Blacks and the need to acquire adaptive capacity to respond to stressful environments may be beneficial to cognitive functioning in later adulthood. Protective factors acquired earlier in the life course may also contribute to cognitive functioning at older ages for Black adults, though empirical studies are limited [50]. Therefore, more research is needed to better understand how psychosocial factors in the context of adverse neighborhood conditions may be associated with better cognitive functioning among older Black adults in spite of being low SES. More research is also needed to understand whether the findings from this study are consistent across other measures of SES (e.g., parental education, income, wealth) in order to determine if education poses a unique effect on cognitive functioning for Black adults.

We acknowledge several limitations in our study. First, because this is a cross-sectional study, we caution against making causal inferences based on these findings. Our primary objective was to document the relationship between perceived neighborhood characteristics and cognitive functioning, rather than to evaluate changes in cognitive functioning, across the intersections of race/ethnicity, gender, and education. Next, while our analyses controlled for nativity, future research should explicitly examine whether and the extent to which nativity intersects with other aspects of social identity, particularly among Mexican American older adults. As our study included self-reported indicators of socioeconomic, psychosocial, behavioral, and health characteristics, we cannot exclude the possibility of recall or other forms of bias in these measures. Finally, we remain cautious in our interpretation of the results of this study due to the possibility of selective mortality, which may be particularly salient among the most disadvantaged groups (e.g., racial/ethnic minorities, less educated individuals).

Given the current findings, we propose future research directions to further shape understanding of how neighborhood characteristics are associated with cognitive functioning, particularly among Black and Mexican older adults. First, statistical analyses can determine if the relationship between neighborhood perceptions and cognitive functioning differs between Black and Mexican adults. Thus, this will determine if the residential context plays a similar role in cognition for racial and ethnic minorities, or if these groups experience unique pathways that contribute to documented cognitive functioning disparities compared to Whites. Due to variation in residential segregation and neighborhood contexts across the US, future research should consider US region and urbanicity to further understand how geographic context influences older minority adults’ cognitive functioning. Our findings, in addition to limited research specifically focusing on Mexican older adults, also reveal the necessity to consider nativity and language as possible contributing factors to cognitive functioning among Mexican adults. Moreover, studies should incorporate neighborhood context and SES at multiple points across the life course. Coupled with measuring cognitive functioning over time, longitudinal research designs informed by a life course framework will improve our understanding of how trajectories of cognitive aging unfold across adulthood. Lastly, research is needed that expands upon a biopsychosocial framework for understanding cognitive health disparities, including examination of biomarkers associated with the stress process that may underlie risk for poor cognitive functioning [7].

## 5. Conclusions

Overall, the present study contributes to the literature on racial/ethnic disparities in cognitive outcomes by considering neighborhood factors as a determinant of cognitive functioning using a nationally representative sample of older adults. We applied intersectionality theory to our methodological approach to elucidate how race/ethnicity, gender, and education pattern the association between neighborhood characteristics and cognitive functioning. Our findings further extend the literature by including measures of perceived neighborhood characteristics related to disorder and social cohesion. From a chronic stress perspective, individuals’ perceptions of their environment may be indicative of biopsychosocial processes underlying cognitive functioning. While we find that negative neighborhood perceptions are related to worse cognitive functioning for older adults, this relationship differs according to education level across racial/ethnic groups. Thus, this research expands our understanding of the association between neighborhood environments and older adults’ cognitive health and further highlights heterogeneity by race/ethnicity, gender, and education in the physical and psychosocial mechanisms linking neighborhoods to cognitive functioning.

## Figures and Tables

**Table 1:** Weighted Characteristics of Study Participants as Means (SE) and Proportions, HRS 2010–2016 (*n* = 8023).

	Overall (n = 8023)	White (n = 6616)	Black (n = 1044)	Mexican (n = 363)
Cognitive functioning	22.12 (0.11)	22.54 (0.11)	18.85 (0.19) *	19.20 (0.37) *
Neighborhood Characteristics				
(Un)safety	2.44 (0.03)	2.35 (0.03)	3.27 (0.06) *	2.96 (0.12) *
(Un)cleanliness	2.32 (0.03)	2.23 (0.02)	3.09 (0.06) *	2.84 (0.14) *
(Dis)cohesion	2.38 (0.02)	2.31 (0.02)	3.03 (0.05) *	2.78 (0.10) *
Sociodemographic Characteristics				
Age	74.18 (0.16)	74.34 (0.18)	73.22 (0.33) *	72.28 (0.37) *
Foreign-born	5.48%	4.22%	4.99%	38.42% *
Woman	56.00%	55.52%	59.97% *	59.21%
Education (years)	13.00 (0.07)	13.28 (0.06)	11.92 (0.12) *	8.66 (0.37) *
Income ($ thousands)	62.74 (2.16)	66.32 (2.36)	37.04 (1.58) *	31.64 (1.63) *
Wealth ($ thousands)	574.42 (29.80)	627.15 (31.83)	162.96 (9.16) *	192.29 (19.14) *
Married/partnered	61.32%	63.05%	40.69% *	65.33%
Behavioral Risk Factors				
Smoking status				
Never smoker	42.57%	42.63%	41.03%	44.55%
Former smoker	48.74%	49.10%	46.60%	44.47%
Current smoker	8.69%	8.27%	12.37% *	10.97%
Alcohol use				
No consumption	63.73%	62.00%	77.39% *	75.70% *
Moderate consumption	30.70%	32.51%	17.46% *	15.59% *
Heavy consumption	5.57%	5.48%	5.15%	8.71%
Moderate/vigorous physical activity				
Never	22.09%	21.49%	28.71% *	22.07%
Sometimes	25.44%	24.79%	30.08% *	31.34% *
Frequent	52.46%	53.72%	41.21% *	46.58% *
Health Characteristics				
Obese	30.80%	29.69%	41.31% *	34.53%
Number of chronic conditions	2.29 (0.02)	2.27 (0.02)	2.56 (0.05) *	2.26 (0.07)
CES-D ≥ 3	17.24%	16.55%	20.36% *	27.35% *

Abbreviations: HRS, Health and Retirement Study; SE, standard error; CESD-D, Center for Epidemiological Studies Depression Scale. * indicates significant difference from White group at *p* < 0.05.

**Table 2:** Linear Regression Models of Associations between Cognitive Functioning and Perceived Neighborhood Characteristics by Gender and Years of Education among non-Hispanic White Older Adults (*n* = 6616).

	Unsafe	Unclean	Social Discohesion
Variable	b (SE)	b (SE)	b (SE)
Neighborhood Characteristic	−0.51 * (0.20)	−0.58 ** (0.20)	−0.95 ** (0.27)
Woman	1.43 *** (0.18)	1.33 *** (0.22)	1.11 *** (0.20)
Education	0.40 *** (0.04)	0.39 *** (0.04)	0.33 *** (0.05)
Woman x Neighborhood			
Characteristic	−0.06 (0.06)	−0.02 (0.08)	0.07 (0.07)
Education x Neighborhood			
Characteristic	0.03 * (0.01)	0.04 ** (0.01)	0.06 ** (0.02)
Constant	25.5 *** (1.07)	25.6 *** (1.03)	26.5 *** (1.28)
*R* ^2^	0.31	0.31	0.31
*F* (22, 6593)	128.43 ***	127.36 ***	128.17 ***

Note: Models control for age, wave, nativity, partnership status, ln income, ln wealth, obesity, physical activity, smoking status, alcohol use, number of chronic conditions, and depression. * *p* < 0.05; ** *p* < 0.01; *** *p* < 0.01.

**Table 3:** Linear Regression Models of Associations between Cognitive Functioning and Perceived Neighborhood Characteristics by Gender and Years of Education among non-Hispanic Black Older Adults (*n* = 1044).

	Unsafe	Unclean	Social Discohesion
Variable	b (SE)	b (SE)	b (SE)
Neighborhood Characteristic	0.48 (0.38)	0.36 (0.38)	0.57 (0.42)
Woman	0.18 (0.69)	0.80 (0.64)	0.99 (0.71)
Education	0.82 *** (0.11)	0.79 *** (0.11)	0.82 *** (0.12)
Woman x Neighborhood			
Characteristic	0.14 (0.18)	−0.05 (0.16)	−0.13 (0.20)
Education x Neighborhood			
Characteristic	−0.05 ^†^ (0.01)	−0.05 ^†^ (0.03)	−0.06 ^†^ (0.03)
Constant	15.2 *** (1.07)	15.6 *** (3.25)	14.7 *** (3.32)
*R* ^2^	0.37	0.37	0.37
*F* (22, 1021)	24.67 ***	24.51 ***	24.47 ***

Note: Models control for age, wave, nativity, partnership status, log income, log wealth, obesity, physical activity, smoking status, alcohol use, number of chronic conditions, and depression. ^†^ *p* < 0.1; *** *p* < 0.01.

**Table 4:** Linear Regression Models of Associations between Cognitive Functioning and Perceived Neighborhood Characteristics by Gender and Years of Education among Mexican Older Adults (*n* = 363).

	Unsafe	Unclean	Social Discohesion
Variable	b (SE)	b (SE)	b (SE)
Neighborhood Characteristic	0.36 * (0.13)	−0.07 (0.28)	0.22 (0.22)
Woman	0.001 (0.20)	−0.18 (0.97)	0.20 (0.84)
Education	0.40 *** (0.04)	0.28 *** (0.06)	0.34 *** (0.05)
Gender x Neighborhood			
Characteristic	−0.06 (0.06)	−0.07 (0.31)	−0.19 (0.26)
Education x Neighborhood			
Characteristic	−0.01 (0.02)	0.04 * (0.01)	0.01(0.02)
Constant	31.0 (3.40)	32.2 *** (3.50)	31.5 *** (3.41)
*R* ^2^	0.40	0.40	0.40
*F* (22, 340)	8.08 ***	8.00 ***	8.02 ***

Note: Models control for age, wave, nativity, partnership status, log income, log wealth, obesity, physical activity, smoking status, alcohol use, number of chronic conditions, and depression. * *p* < 0.05; *** *p* < 0.01.

## Data Availability

Data for this study were obtained from the publically available Health and Retirement Study data files at https://hrs.isr.umich.edu/data-products (accessed on 5 March 2021).

## Supplementary Materials

The following are available online at https://www.mdpi.com/1660-4601/18/5/2661/s1, Table S1: Skewness and Kurtosis for Key Continuous Variables (*n* = 8023), Table S2: Linear Re-gression Models of Associations between Cognitive Functioning and Perceived Neighborhood Characteristics by Gender and Years of Education among non-Hispanic White Older Adults (*n* = 6616), Table S3: Linear Regression Models of Associations between Cognitive Functioning and Perceived Neighborhood Characteristics by Gender and Years of Education among non-Hispanic Black Older Adults (*n* = 1044), Table S4: Linear Regression Models of Associations between Cogni-tive Functioning and Perceived Neighborhood Characteristics by Gender and Years of Education among Mexican Older Adults (*n* = 363). Figure S1: Perceived unsafe neighborhood by years of education predicting cognitive functioning among non-Hispanic white older adults (*n* = 6616), Figure S2: Perceived unsafe neighborhood by years of education predicting cognitive functioning among non-Hispanic Black Older Adults (*n* = 1044), Figure S3: Perceived Social decohesion by years of education predicting cognitive functioning among non-Hispanic white older adults (*n* = 6616), Figure S4: Perceived Social decohesion by years of education predicting cognitive function-ing among non-Hispanic Black Older Adults (*n* = 1044).
