# Supplementary material for: Perceived Neighborhood Characteristics and Cognitive Functioning among Diverse Older Adults: An Intersectional Approach
Source: Int J Environ Res Public Health. 2021 Mar 6;18(5):2661. doi: 10.3390/ijerph18052661 (PMC7967341; doi:10.3390/ijerph18052661)
Supplement: Supplementary file 1 [file ijerph-18-02661-s001.pdf]

Supplemental Table S1. Skewness and Kurtosis for Key Continuous Variables (*n* = 8023).

Variable	Skewness	Kurtosis
Cognitive Functioning	-0.64	3.75
Years of Education	-0.83	4.93
Neighborhood Characteristic		
Unsafe	1.10	3.43
Unclean	1.23	3.89
Discohesive	1.19	4.02
Age	0.59	2.80
Number of Chronic Conditions	0.31	2.80
Ln Income	15.92	508.17
Ln Wealth	9.88	192.73

Supplemental Table S2. Linear Regression Models of Associations between Cognitive Functioning and Perceived Neighborhood Characteristics by Gender and Years of Education among non-Hispanic White Older Adults (*n* = 6616).

	Unsafe	Unclean	Social Discohesion
**Variable**	**b (SE)**	**b (SE)**	**b (SE)**
Neighborhood Characteristic	-0.51* (0.20)	-0.58** (0.20)	-0.95** (0.27)
Woman	1.43*** (0.18)	1.33*** (0.22)	1.11*** (0.20)
Education	0.40*** (0.04)	0.39*** (0.04)	0.33*** (0.05)
Woman x Neighborhood Characteristic	-0.06 (0.06)	-0.02 (0.08)	0.07 (0.07)
Education x Neighborhood Characteristic	0.03* (0.01)	0.04** (0.01)	0.06** (0.02)
Age	-0.19*** (0.01)	-0.20*** (0.01)	-0.20*** (0.01)
Wave			
2010 (reference)			
2012	-0.02 (0.10)	-0.03 (0.10)	-0.02 (0.10)
2014	-0.57 (0.25)	-0.57* (0.25)	-0.56* (0.25)
2016	-0.52 (0.34)	-0.53 (0.33)	-0.52 (0.34)
Foreign Born	-0.65* (0.28)	-0.64* (0.28)	-0.65* (0.28)
Married/Partnered	-0.35* (0.13)	-0.35* (0.13)	-0.35* (0.13)
Ln Income	1.14*** (0.18)	1.14*** (0.18)	1.16*** (0.18)
Ln Wealth	0.03 (0.03)	0.04 (0.03)	0.03 (0.03)
Obese	0.48*** (0.13)	0.47*** (0.13)	0.48*** (0.13)
Moderate/Vigorous Activity			
Never (reference)			
Sometimes	0.64*** (0.13)	0.64*** (0.13)	0.63*** (0.13)
Often	0.76*** (0.13)	0.77*** (0.13)	0.75*** (0.13)
Smoking Status			
Never Smoked (reference)			
Former Smoker	0.14 (0.13)	0.14 (0.13)	0.14 (0.13)
Current Smoker	-0.41† (0.24)	-0.41† (0.23)	-0.41† (0.24)
Alcohol Use			
No Consumption (reference)			
Moderate Consumption	0.61*** (0.11)	0.62*** (0.11)	0.63*** (0.11)
Heavy Consumption	0.68** (0.23)	0.70** (0.23)	0.68** (0.23)
Total Chronic Conditions	-0.11* (0.04)	-0.11* (0.04)	-0.11* (0.04)
Depressed	-0.84*** (0.16)	-0.86*** (0.16)	-0.82*** (0.15)
Constant	25.5*** (1.07)	25.6*** (1.03)	26.5*** (1.28)
R^2	0.31	0.31	0.31
F (22, 6,593)	128.43***	127.36***	128.17***

†p < 0.1; *p < 0.05; **p < 0.01; ***p < 0.01

Supplemental Table S3. Linear Regression Models of Associations between Cognitive Functioning and Perceived Neighborhood Characteristics by Gender and Years of Education among non-Hispanic Black Older Adults (*n* = 1044).

	Unsafe	Unclean	Social Discohesion
**Variable**	**b (SE)**	**b (SE)**	**b (SE)**
Neighborhood Characteristic	0.48 (0.38)	0.36 (0.38)	0.57 (0.42)
Woman	0.18 (0.69)	0.80 (0.64)	0.99 (0.71)
Education	0.82*** (0.11)	0.79*** (0.11)	0.82*** (0.12)
Woman x Neighborhood Characteristic	0.14 (0.18)	-0.05 (0.16)	-0.13 (0.20)
Education x Neighborhood Characteristic	-0.05† (0.01)	-0.05† (0.03)	-0.06† (0.03)
Age	-0.16*** (0.03)	-0.16*** (0.03)	-0.16*** (0.03)
Wave			
2010 (reference)			
2012	0.20 (0.40)	0.18 (0.39)	0.18 (0.39)
2014	-0.34 (0.57)	-0.33 (0.56)	-0.33 (0.55)
2016	-0.63 (0.53)	-0.63 (0.52)	-0.54 (0.54)
Foreign Born	-0.42 (0.81)	-0.38 (0.77)	-0.22 (0.78)
Married/Partnered	-0.11 (0.35)	-0.12 (0.36)	-0.14 (0.34)
Ln Income	1.04*** (0.22)	1.04*** (0.22)	1.08*** (0.22)
Ln Wealth	0.11 (0.09)	0.11 (0.09)	0.11 (0.10)
Obese	0.76** (0.28)	0.75* (0.28)	0.76 (0.28)
Moderate/Vigorous Activity			
Never (reference)			
Sometimes	0.94* (0.40)	0.94* (0.40)	0.93* (0.40)
Often	0.53 (0.37)	0.54 (0.37)	0.57 (0.37)
Smoking Status			
Never Smoked (reference)			
Former Smoker	-0.23 (0.27)	-0.25 (0.27)	-0.26 (0.27)
Current Smoker	-0.34 (0.48)	-0.29 (0.49)	-0.32 (0.48)
Alcohol Use			
No Consumption (reference)			
Moderate Consumption	0.94* (0.36)	0.95* (0.36)	0.93* (0.36)
Heavy Consumption	-0.16 (0.75)	-0.21 (0.74)	-0.30 (0.76)
Total Chronic Conditions	0.07 (0.14)	0.09 (0.14)	0.10 (0.15)
Depressed	-1.53*** (0.30)	-1.55*** (0.31)	-1.53*** (0.31)
Constant	15.2*** (1.07)	15.6*** (3.25)	14.7*** (3.32)
R^2	0.37	0.37	0.37
F (22, 1,021)	24.67***	24.51***	24.47***

†p < 0.1; *p < 0.05; **p < 0.01; ***p < 0.01

Supplemental Table S4. Linear Regression Models of Associations between Cognitive Functioning and Perceived Neighborhood Characteristics by Gender and Years of Education among Mexican Older Adults (*n* = 363).

	Unsafe	Unclean	Social Discohesion
**Variable**	**b (SE)**	**b (SE)**	**b (SE)**
Neighborhood Characteristic	0.36* (0.13)	-0.07 (0.28)	0.22 (0.22)
Woman	0.001 (0.20)	-0.18 (0.97)	0.20 (0.84)
Education	0.40*** (0.04)	0.28*** (0.06)	0.34*** (0.05)
Gender x Neighborhood Characteristic	-0.06 (0.06)	-0.07 (0.31)	-0.19 (0.26)
Education x Neighborhood Characteristic	-0.01 (0.02)	0.04* (0.01)	0.01(0.02)
Age	-0.23*** (0.04)	-0.22*** (0.04)	-0.23*** (0.04)
Wave			
2010 (reference)			
2012	-0.001 (0.29)	0.02 (0.30)	-0.03 (0.32)
2014	0.77 (0.60)	0.76 (0.62)	0.72 (0.60)
2016	0.13 (0.57)	0.30 (0.55)	0.25 (0.57)
Foreign Born	0.33 (0.38)	0.35 (0.37)	0.31 (0.39)
Married/Partnered	-0.18 (0.32)	-0.01 (0.35)	-0.10 (0.32)
Ln Income	-0.15† (0.08)	-0.21* (0.08)	-0.17* (0.08)
Ln Wealth	0.23** (0.06)	0.21** (0.06)	0.23** (0.05)
Obese	-1.32** (0.37)	-1.39** (0.37)	-1.39** (0.33)
Moderate/Vigorous Activity			
Never (reference)			
Sometimes	1.30** (0.40)	1.23* (0.42)	1.32** (0.42)
Often	0.68 (0.57)	0.74 (0.61)	0.75 (0.62)
Smoking Status			
Never Smoked (reference)			
Former Smoker	0.58 (0.38)	0.49 (0.40)	0.58 (0.41)
Current Smoker	-1.36† (0.64)	-1.40* (0.61)	-1.35* (0.57)
Alcohol Use			
No Consumption (reference)			
Moderate Consumption	2.29*** (0.27)	2.29*** (0.31)	2.29*** (0.33)
Heavy Consumption	-0.36 (0.32)	-0.33 (0.27)	-0.44 (0.33)
Total Chronic Conditions	-0.10 (0.17)	-0.12 (0.16)	-0.11 (0.16)
Depressed	-0.72 (0.55)	-0.68 (0.52)	-0.70 (0.55)
Constant	31.0 (3.40)	32.2*** (3.50)	31.5*** (3.41)
R^2	0.40	0.40	0.40
F (22, 340)	8.08***	8.00***	8.02***

†p < 0.1; *p < 0.05; **p < 0.01; ***p < 0.01

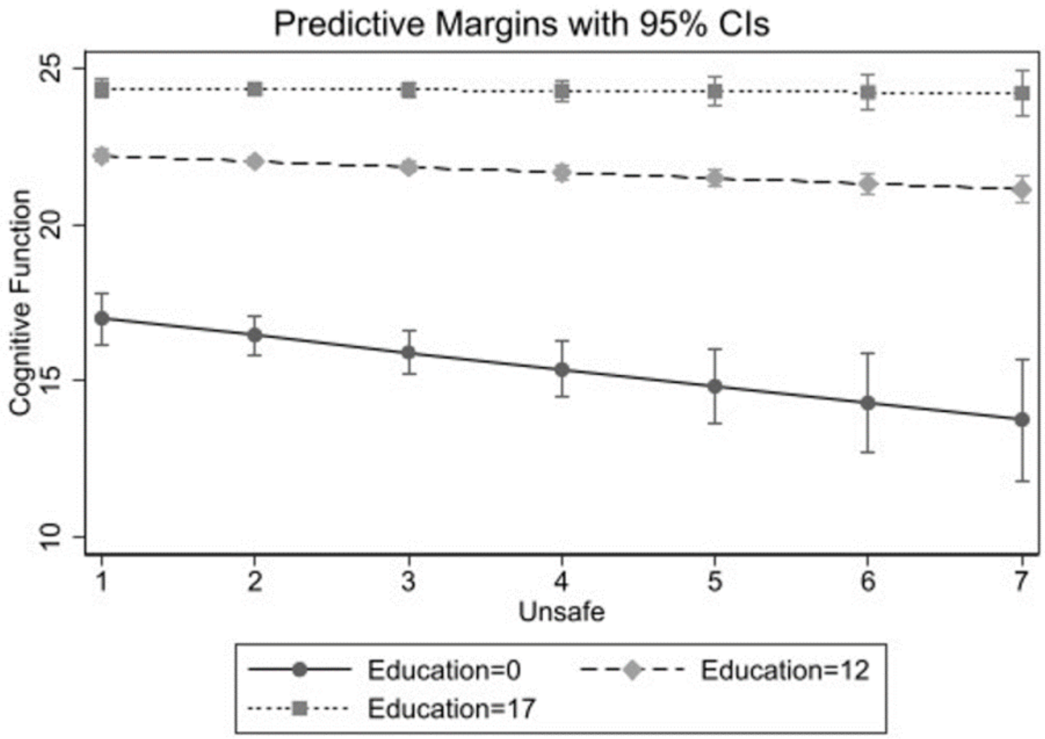

Supplemental Figure 1. Perceived Unsafe Neighborhood by Years of Education Predicting Cognitive Functioning among Non-Hispanic White Older Adults (n=6,616).

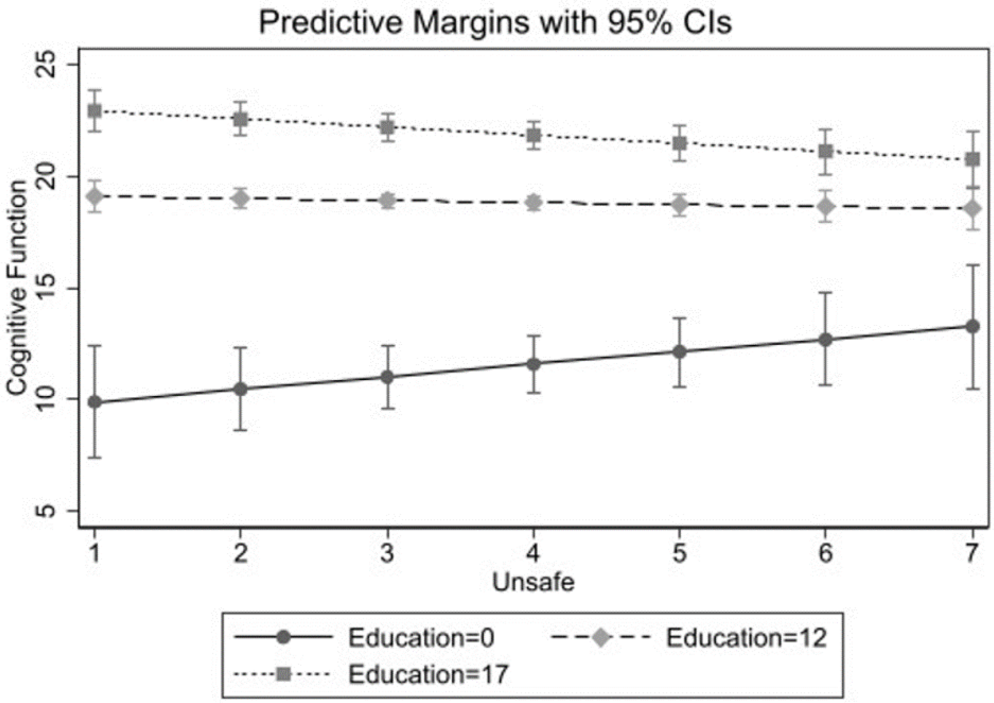

Supplemental Figure 2. Perceived Unsafe Neighborhood by Years of Education Predicting Cognitive Functioning among Non-Hispanic Black Older Adults (n=1,044).

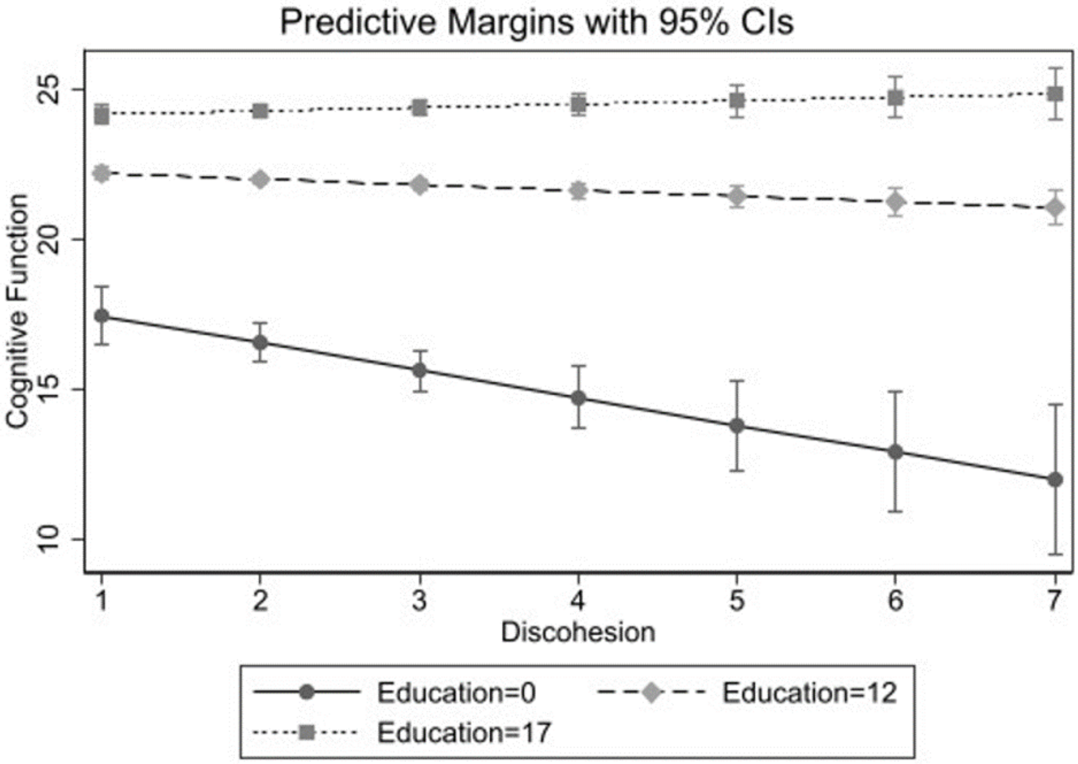

Supplemental Figure 3. Perceived Social Discohesion by Years of Education Predicting Cognitive Functioning among Non-Hispanic White Older Adults (n=6,616).

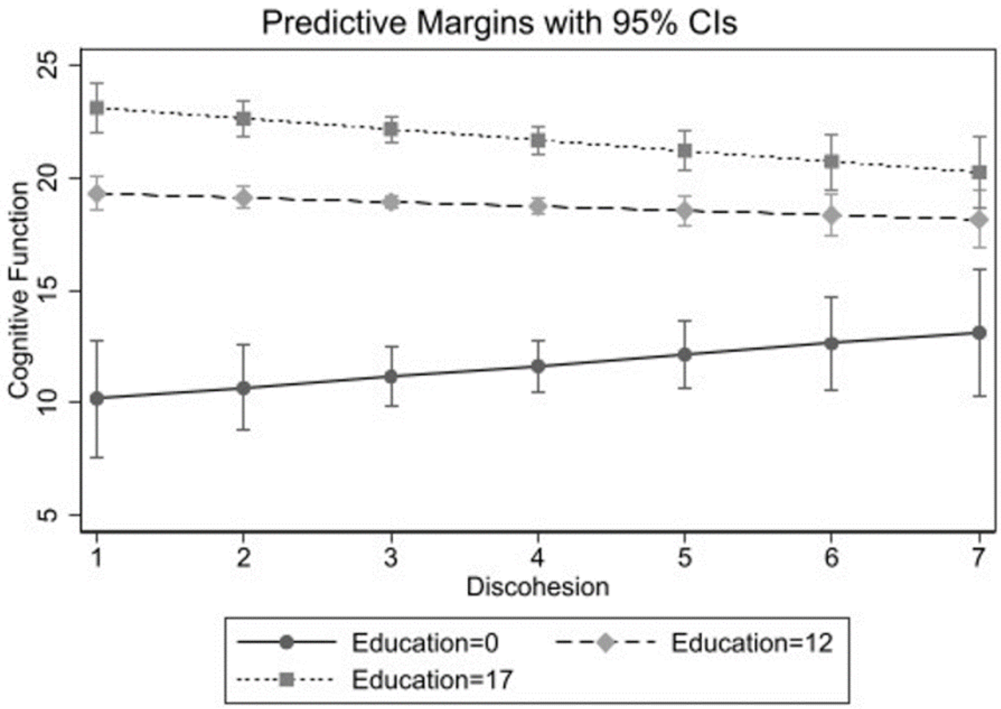

Supplemental Figure 4. Perceived Social Discohesion by Years of Education Predicting Cognitive Functioning among Non-Hispanic Black Older Adults (n=1,044).
